# Lutein Attenuates Synovial Hyperplasia, Cartilage Loss, and Bone Erosion by Suppressing Inflammatory Pathways in Rheumatoid Arthritis

**DOI:** 10.7150/ijms.135369

**Published:** 2026-06-10

**Authors:** Chi-Jen Chang, Tsung-Ming Chang, Ying-Sui Sun, Ji-Fan Lin, Skye Hsin-Hsien Yeh, Ju-Fang Liu

**Affiliations:** 1 School of Medicine, Fu Jen Catholic University, New Taipei City 242062, Taiwan.; 2 Division of Pediatric Surgery, Shin Kong Wu Ho-Su Memorial Hospital, Taipei 111045, Taiwan.; 3 School of Dental Technology, College of Oral Medicine, Taipei Medical University, Taipei 110031, Taiwan.; 4 Translational Medicine Center, Shin Kong Wu Ho-Su Memorial Hospital, Taipei 111045, Taiwan.; 5 School of Medicine, National Defense Medical Center, Taipei 114824, Taiwan.; 6 School of Oral Hygiene, College of Oral Medicine, Taipei Medical University, Taipei 110031, Taiwan.; 7 Department of Medical Research, China Medical University Hospital, China Medical University, Taichung 406040, Taiwan.

**Keywords:** rheumatoid arthritis, lutein, RNA sequencing, collagen antibody-induced arthritis (CAIA), fibroblast-like synovial cell

## Abstract

Lutein is a bioactive carotenoid best known for its role in retinal protection, but emerging evidence suggests that it may also exert anti-inflammatory activity in chronic inflammatory disorders. In this study, we investigated the anti-inflammatory and joint-protective effects of lutein in rheumatoid arthritis (RA)-related experimental models. Transcriptomic profiling was performed in MH7A fibroblast-like synovial cells treated with lutein, followed by Gene Ontology (GO), Kyoto Encyclopedia of Genes and Genomes (KEGG), and gene set enrichment analysis (GSEA). The results showed that lutein regulated inflammation-related transcriptional programs, particularly pathways associated with cytokine signaling and inflammatory responses. In TNF-α-activated MH7A cells, lutein suppressed inflammatory cytokine production and inhibited cell proliferation. The *in vivo* effects of lutein were further evaluated in a collagen antibody-induced arthritis (CAIA) mouse model. Mice were assigned to control, CAIA, and lutein-treated CAIA groups, and disease progression was assessed by monitoring body weight, paw thickness, and arthritis scores. Lutein treatment significantly attenuated arthritis severity and joint inflammation without affecting body weight. Micro-computed tomography demonstrated reduced bone erosion and osteophyte formation in lutein-treated CAIA mice. Histological analyses using hematoxylin and eosin and Safranin O staining further showed that lutein alleviated synovial hyperplasia and cartilage destruction. These findings indicate that lutein exerts anti-inflammatory and joint-protective effects in experimental models relevant to RA and support further mechanistic and preclinical investigation of lutein as a bioactive natural compound in inflammatory joint disease.

## Introduction

Rheumatoid arthritis (RA) is an autoimmune disorder characterized by persistent inflammation and synovial proliferation within symmetrical joints, resulting in pain, swelling, cartilage damage, and bone erosion 1, 2. Although nonsteroidal anti-inflammatory drugs (NSAIDs), corticosteroids, disease-modifying antirheumatic drugs (DMARDs), and biologic agents constitute the current standard of care for RA 3, 4, the use of these medications remains limited by incomplete symptom control and treatment-related adverse effects, including increased susceptibility to infection, hepatotoxicity, and osteoporosis 5, 6. Despite these therapeutic options, many patients continue to experience residual disease activity and treatment-related toxicities. Thus, there remains an unmet need for strategies that more effectively balance efficacy with long-term safety in RA management.

The pathogenesis of RA involves a complex interplay between cytokines and fibroblast-like synovial cells 7-9. Pro-inflammatory cytokines such as tumor necrosis factor-alpha (TNF-α), interleukin-1β (IL-1β), and interleukin-6 (IL-6) are key mediators of inflammation that drive the chronic immune responses characteristic of RA 10-12. These cytokines stimulate synovial fibroblasts, which are integral to the pathology of RA, to proliferate and secrete additional pro-inflammatory cytokines. These cytokines then recruit immune cells for infiltration and vascular proliferation and further amplify the inflammatory response 13-16. Stimulated synovial fibroblasts also contribute to the destruction of cartilage and bone by producing and secreting enzymes (matrix metalloproteinases, MMPs) that degrade extracellular matrix components 17, 18. This vicious cycle of cytokine production and synovial fibroblast activation perpetuates inflammation as well as leads to joint damage and deformities associated with RA 19, 20, emphasizing the critical roles that both cytokines and synovial fibroblasts play in the progression of this disease.

As a naturally occurring carotenoid found in fruits and vegetables, lutein has recognized antioxidant and anti-inflammatory properties and has been investigated in several inflammation-associated conditions 21, 22. In addition to its established role in ocular health, lutein has been reported to modulate inflammatory signaling and reduce the production of mediators such as TNF-α and IL-6 23. Experimental studies have further shown protective effects of lutein in models of liver and lung injury and in bone-related disorders linked to oxidative stress 24, 25. Serum lutein levels have also been inversely associated with cardiovascular disease risk 26. Because oxidative stress and inflammatory signaling contribute to RA pathogenesis 8 , lutein represents a bioactive natural product of interest for mechanistic evaluation in RA; however, its effects in this setting remain insufficiently characterized.

In this study, we aimed to evaluate the anti-inflammatory and joint-protective potential of lutein in rheumatoid arthritis and to investigate the molecular basis underlying its bioactivity. To this end, transcriptome profiling was employed to explore RA-related pathways potentially modulated by lutein, followed by *in vitro* studies in MH7A synoviocytes and *in vivo* validation in a collagen antibody-induced arthritis mouse model. Histological and immunohistochemical analyses were further used to assess inflammatory alterations in joint tissues. Collectively, this study was designed to provide molecular and biological insight into lutein as a bioactive natural product in experimental inflammatory joint disease.

## Material and Methods

### Materials

The rabbit polyclonal antibodies against IL-1β (sc-52012) and IL-6 (sc-57315) were purchased from Santa Cruz Biotechnology (TX, USA). All other reagents were purchased from Sigma-Aldrich (St. Louis, MO, USA).

### Cell cultures

MH7A cells (Riken Cell Bank, Ibaraki, Japan) were grown on 10 cm culture dishes in 95% air/5% CO_2_ with RPMI 1640 (Life Technologies, Grand Island, NY, USA) supplemented with 10% heat-inactivated fetal bovine serum, 2 mM glutamine, 100 U/ml penicillin, and 100 μg/ml streptomycin (pH adjusted to 7.6). Data are expressed from four independent experiments (n = 4).

### RNA sequencing analysis

To investigate the effects of lutein on gene expression in MH7A cells, cells were cultured in medium with or without lutein (10 μM) for 24 h 23, 27, 28. Three biological replicates were included in both the control and lutein-treated groups. Total RNA was extracted using the Easy-BLUE Total RNA Extraction Kit (iNtRON Biotechnology, Seongnam-si, Gyeonggi-do, Korea) and submitted to Biotools Co., Ltd. (Taiwan) for RNA-sequencing analysis. Sequencing was performed on the NovaSeq 6000 platform (Illumina, USA) with paired-end 150 bp reads. Reads were aligned using HISAT2, and expression data were normalized using relative log expression (RLE) and trimmed mean of M-values (TMM). Differentially expressed genes were defined using an adjusted *p*-value < 0.05 and an absolute log_2_ fold change ≥ 0.807. Gene set enrichment analysis (GSEA) was based on the original RNA-seq analysis report generated from the ranked transcriptomic data. For Gene Ontology (GO) and Kyoto Encyclopedia of Genes and Genomes (KEGG) enrichment analyses, downregulated DEGs identified using the revised threshold were further analyzed using the STRING database 29. Enriched terms were filtered using a false discovery rate (FDR) cutoff of ≤ 0.05, grouped by similarity with a threshold of 0.8, and ranked by signal score. Gene counts were also displayed for the enriched GO terms and KEGG pathways.

### Quantitative real-time PCR

RNA was extracted using the Easy-Blue Total RNA Extraction Kit. First-strand cDNA was obtained using a qPCRBIO cDNA Synthesis Kit (Cat. No. PB30.11-10, PCR Biosystems, London, England, UK). mRNA expression of human IL-1β, IL-6, and GAPDH was examined by qPCR. A KAPA SYBR FAST qPCR Master Mix (2X) kit (Sigma-Aldrich) on a CFX Connect real-time PCR detection system (Bio-Rad, Hercules, CA, USA) was used according to the manufacturer's instructions. All primers were purchased from Sigma-Aldrich, and GAPDH was used as an internal control. Gene expression levels were determined using the 2^-ΔΔCt^ method.

### Cell viability assay

Cells (1 × 10^4^) were seeded into each well of a 48-well plate overnight. On the following day, the medium was replenished with fresh media containing various concentrations of lutein. After 24 h, 10 μl of CCK-8 (Sigma-Aldrich) was added to each well followed by a further incubation for 4 h. The microplate was measured at an absorbance of 450 nm using a VARIOSKAN LUX microplate reader.

### CAIA induction and treatment

Seven-week-old male BALB/c mice purchased from Lasco (BioLASCO Co., Ltd, Taipei, Taiwan) were randomized into three groups and maintained in pathogen-free conditions. A week before the induction of collagen type II antibody-induced arthritis (CAIA) in groups 2 and 3, corn oil was administered daily to mice in groups 1 and 2 via oral gavage while lutein (40 mg/kg) dissolved in corn oil was fed daily to mice in group 3 until the end of the experiment 30. CAIA was induced in groups 2 and 3 as follows: on day 0, mice were intravenously injected with 1 mg of a 5-clone monoclonal antibody cocktail (Chondrex); and on day 3, mice were intraperitoneally injected with LPS (50 μg/100 μL) to exacerbate the systemic inflammatory response. Body weight, paw thickness, ankle thickness, and arthritis score were evaluated on days 4, 7, 10, 13, 16, 19, and 22. All mice were sacrificed on day 22, and both blood and limbs were collected for further studies. All animal experiments were conducted in strict compliance with the protocols approved by the Institutional Ethics Committee of Shin Kong Wu Ho-Su Memorial Hospital (IACUC Approval No: 112NSTCIACUC007).

### Evaluation of arthritis

Evaluation of arthritis was conducted according to previously published methods 31, 32. The severity of arthritis was evaluated in each limb on a scale of 0 to 4 (0 = no obvious differences in appearance vs. healthy mice; 1 = one or two toes inflamed and swollen without apparent swelling of paw or ankle; 2 = three or more toes inflamed and swollen, or mild paw swelling; 3 = swelling of entire paw; and 4 = severe swelling of entire paw and all toes). The maximum score for each mouse was 16.

### Paw volume meter

The efficacy of lutein was evaluated by measuring the changes in paw volume during the swelling process. The forelimbs and hindlimbs of the mice were placed in the paw volume meter to record the increase in water volume. Corrections were made after each measurement, and the average of three trials was obtained.

### Micro-CT

Images of *ex vivo* trabecular bone microarchitecture were acquired using micro-computed tomography (MILabs, Utrecht, Netherlands). The micro-CT imaging parameters were as follows: X-ray voltage of 80 kVp, anode current of 500 μA, and exposure time of 300-350 milliseconds for each of the 360 rotational steps. This level of voltage is optimal for establishing high-contrast images of bones with minimal beam hardening. CT images were reconstructed using a Hann filter with a voxel size of 5 µm. The volume of individual samples was measured by drawing regions of interest (ROI) outlining individual samples on each CT slice, summing the areas of the individual ROI measured in all slices, and multiplying it by slice thickness to calculate the sample volume. The images were analyzed using BoneJ (Domander, R., Felder, A. A., & Doube, M. (2021), BoneJ2 - refactoring established research software) 33. The threshold for bone tissue was -100 Hounsfield units (HU). After acquiring the volumes in voxels in cm³ for bone tissue, the bone volume fraction (bone volume/total volume, BV/TV%) and trabecular thickness (Tb. Th μm) of the three experimental groups were calculated. The 3D image stacks were reconstructed from the rotational image projections using reconstruction software (Osiris, Geneva, Switzerland).

### Hematoxylin and eosin staining

Sections (3 μm) were prepared from paraffin-embedded tissues, then deparaffinized in xylene, rehydrated in a graded alcohol series, and washed in deionized water. Nuclei were stained with hematoxylin solution for 10 min. After differentiation with 0.3% acid alcohol, the sections were rinsed in Scott's tap water substitute and incubated in an eosin solution for 2 min to visualize the cytosol. The sections were then observed under a light microscope.

### Safranin O staining

After deparaffinization, 3-μm sections were rehydrated in deionized water. The sections were stained with Weigert's iron hematoxylin solution for 10 minutes. Excess hematoxylin was removed by rinsing in tap water for 10 min. The sections were then stained with 0.05% fast green solution for 5 minutes and quickly rinsed with a 1% acetic acid solution for 10-15 seconds. The cartilage was immediately stained with 0.1% Safranin O solution for 5 minutes and dehydrated using alcohol and xylene. Safranin O-stained sections were scored using the modified Osteoarthritis Research Society International (OARSI) scoring system. The scoring items include cartilage surface regularity (0 to 3), depth of clefts into the cartilage layer (0 to 3), decreased chondrocyte density (0 to 3), and chondrocyte cloning (0 to 3), with the final score ranging from 0 to 12 34.

### Immunohistochemistry

After the sections were deparaffinized, 3-μm sections were rehydrated in deionized water. Following antigen retrieval (boiling on a hotplate at 95-100 °C for 15 min in 10 mM sodium citrate, pH 6.0), the intrinsic peroxidase activity was blocked by incubation with Peroxidase Block (Novolink Polymer Detection Systems, Leica Biosystems, Deer Park, IL, USA). Nonspecific antibody-binding sites were blocked using the Protein Block. Sections were incubated with appropriately diluted primary antibodies specific for IL-1β and IL-6 overnight at 4 °C, followed by washing with PBS-Tween and incubation with a Novolink Polymer for 1 h at room temperature. Stained sections were detected using 3,3'-diaminobenzidine tetrahydrochloride, then counterstained with hematoxylin and observed under a light microscope. IHC results were scored by considering the percentage of positive detection (0-100) using Image J 1.52a and the intensity of staining (0-3+), resulting in a final score ranging from 0 to 300.

### Statistics

The data are presented as mean ± standard deviation. Two groups of data were compared using Student's t-test. More than two groups of data were compared using a one-way analysis of variance followed by the Fisher LSD post-hoc test when appropriate. A *p*-value of < 0.05 was considered statistically significant.

## Results

### Lutein Attenuates Inflammatory Transcriptomic Signatures in MH7A Cells

**Figure 1:**
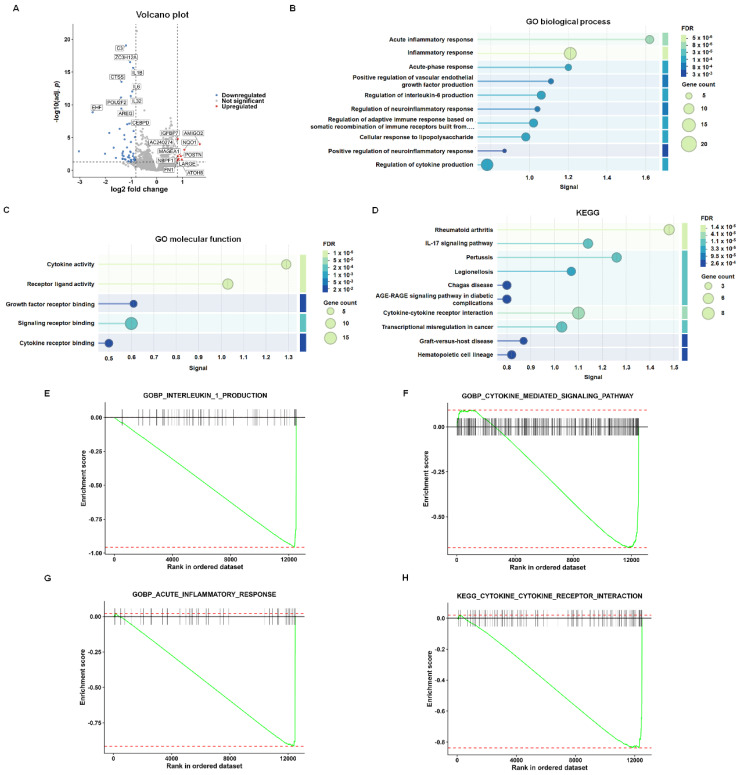
** Lutein suppresses inflammation-related transcriptomic signatures in MH7A cells.** (A) Volcano plot of differentially expressed genes (DEGs) in lutein-treated cells compared with untreated controls. Each dot represents one gene. Red dots indicate significantly upregulated genes, blue dots indicate significantly downregulated genes, and gray dots indicate genes without significant differential expression. (B) Top 10 downregulated Gene Ontology (GO) biological process terms. (C) Top 5 downregulated GO molecular function terms. (D) Top 10 downregulated Kyoto Encyclopedia of Genes and Genomes (KEGG) pathways. (E-H) Gene set enrichment analysis (GSEA) plots for interleukin-1 production, cytokine-mediated signaling pathway, acute inflammatory response, and cytokine-cytokine receptor interaction, respectively. The green line indicates the running enrichment score, black vertical lines indicate gene positions in the ranked list, and the color bar indicates the ranking metric.

To investigate the transcriptomic effects of lutein in MH7A cells, RNA-seq analysis was performed following treatment with lutein (10 μM). The volcano plot showed marked changes in gene expression in lutein-treated cells compared with untreated controls (Fig. 1A). Enrichment analyses of downregulated differentially expressed genes revealed significant association with inflammation-related biological processes, including acute inflammatory response, inflammatory response, regulation of interleukin-6 production, and regulation of cytokine production (Fig. 1B). Downregulated genes were also enriched in cytokine-associated molecular functions, such as cytokine activity, receptor ligand activity, growth factor receptor binding, signaling receptor binding, and cytokine receptor binding (Fig. 1C). KEGG pathway analysis further showed that these downregulated genes were enriched in rheumatoid arthritis, IL-17 signaling pathway, cytokine-cytokine receptor interaction, and other inflammation-related pathways (Fig. 1D). Consistently, GSEA showed negative enrichment of gene sets related to interleukin-1 production, cytokine-mediated signaling pathway, acute inflammatory response, and cytokine-cytokine receptor interaction in lutein-treated cells (Fig. 1E-H). Together, these findings indicate that lutein suppresses inflammation-related transcriptomic programs in MH7A fibroblast-like synovial cells.

### Lutein Reduced Inflammatory Cytokines and Cellular Proliferation in MH7A Cells

**Figure 2:**
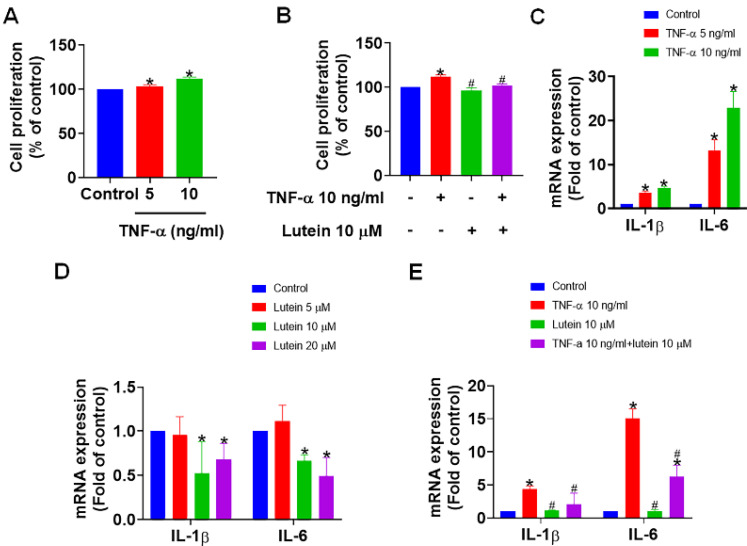
**Lutein reduces synovial fibroblast proliferation and inflammation.** (A) MH7A cells were treated with TNF-α (0, 5, and 10 ng/ml) for 24 h, and cell viability was analyzed by a CCK-8 assay. (B) MH7A cells were pretreated with lutein (10 μM) for 1 h and then treated with TNF-α (0 and 10 ng/ml) for 24 h. Cell viability was analyzed using a CCK-8 assay. (C) qPCR analysis of IL-1β and IL-6 expression in MH7A cells treated with TNF-α (0, 5, and 10 ng/ml) for 24 h. (D) qPCR analysis of IL-1β and IL-6 expression in MH7A cells treated with lutein (0, 5, 10, and 20 μM) for 24 h. (E) MH7A cells were pretreated with lutein (10 μM) for 1 h and then treated with TNF-α (10 ng/ml) for 24 h. The expression of IL-1β and IL-6 was analyzed by qPCR. Untreated cells were used as controls. Results are shown as means ± SD. *p < 0.05 compared to untreated control. #*p* < 0.05 compared to TNF-α.

Given the significant alterations in the expression of genes associated with RA, we next investigated the effects of lutein *in vitro* on inflammation in RA. Treatment of MH7A cells with TNF-α resulted in MH7A cell activation with a corresponding marked increase in cell proliferation, thus exacerbating the inflammatory responses characteristic of RA (Fig. 2A). However, pre-treatment with lutein significantly reversed TNF-α-induced proliferation (Fig. 2B), suggesting an inhibitory role of lutein against synovial cell hyperplasia. Further analysis indicated that TNF-α treatment in MH7A cells elevated the levels of pro-inflammatory cytokines IL-1β and IL-6, which are key mediators of inflammation in RA (Fig. 2C). Remarkably, administering lutein alone attenuated the expression of these cytokines (Fig. 2D), and pre-treatment with lutein prior to TNF-α exposure led to a significant reduction in IL-1β and IL-6 levels (Fig. 2E). These findings indicate that lutein can inhibit inflammatory mediators and suppress synoviocyte proliferation in an experimental cell model relevant to RA.

### Lutein Reduced Arthritis-Associated Index without Affecting Body Weight in CAIA Mice

**Figure 3:**
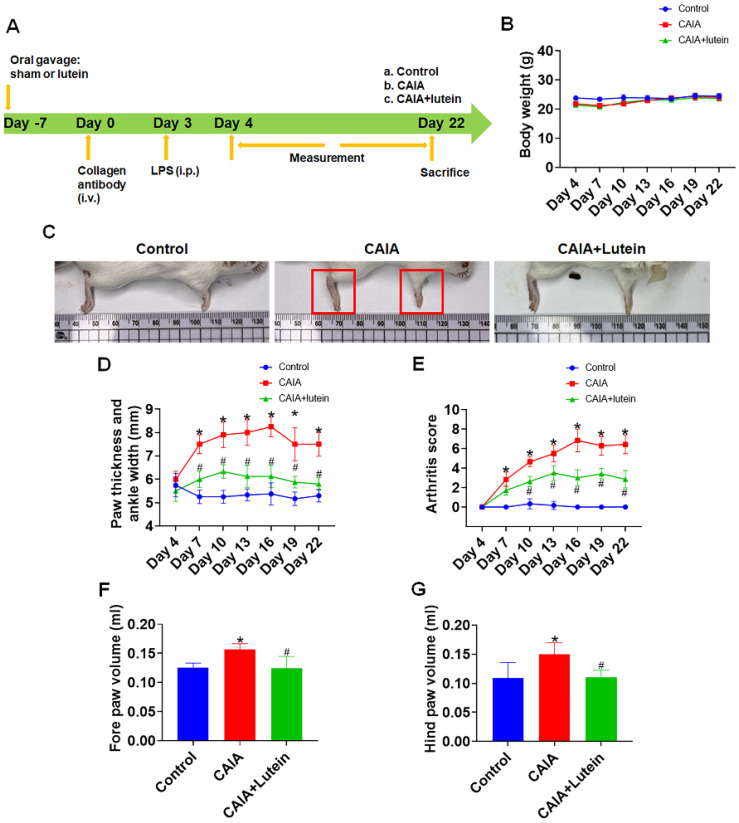
** Lutein alleviates collagen antibody-induced arthritis in mice.** (A) Experimental Design. (B) Mouse body weight during the experiment (n = 4). (C) Representative images of the mouse paws. (D) Quantification of forepaw, hind paw, and ankle thickness (n = 4). (E) The arthritis score was determined based on the level of redness and swelling in the front and rear paws of mice, as well as the count of joint swellings (n = 4). (F)-(G) Paw volume was measured using a paw volume meter in each group at the end of the experiment (n = 4). Untreated mice were used as controls. Results are shown as means ± SD. *p < 0.05 compared to untreated control. #*p* < 0.05 compared to CAIA.

We next utilized an *in vivo* murine CAIA-induced arthritis model to evaluate the *in vivo* effects of lutein in experimental arthritis. Following a one-week acclimatization period, the mice were divided into three distinct groups. The control group received a daily oral gavage of corn oil (day -7), the second group was administered corn oil (day -7) followed by CAIA induction seven days later (day 0), and the third group was treated with a daily oral gavage of lutein (day -7) followed by CAIA induction seven days later (day 0). Daily oral feeding continued until the animals were sacrificed. To exacerbate systemic inflammatory response, LPS was administered on day 3 to the second and third groups. We monitored body weight, joint thickness, and the arthritis index at three-day intervals, starting from day 4 and continuing until the end of the experiment (Fig. 3A). Note that the body weight of mice remained consistent across all groups (Fig. 3B), suggesting that did not affect body weight under these experimental conditions. In the CAIA-corn oil group, the mice exhibited increased paw and ankle redness, thickness, and swelling as well as high overall arthritis scores. Encouragingly, mice that were subjected to CAIA but pre-treated with lutein exhibited a significant reduction in paw and ankle redness, thickness, swelling, and overall arthritis scores compared with mice in the CAIA-corn oil group (Fig. 3C-G). These findings further support the anti-inflammatory and joint-protective effects of lutein in experimental inflammatory arthritis.

### Lutein Protected against Bone Erosion and Osteophyte Formation in CAIA Mice

**Figure 4:**
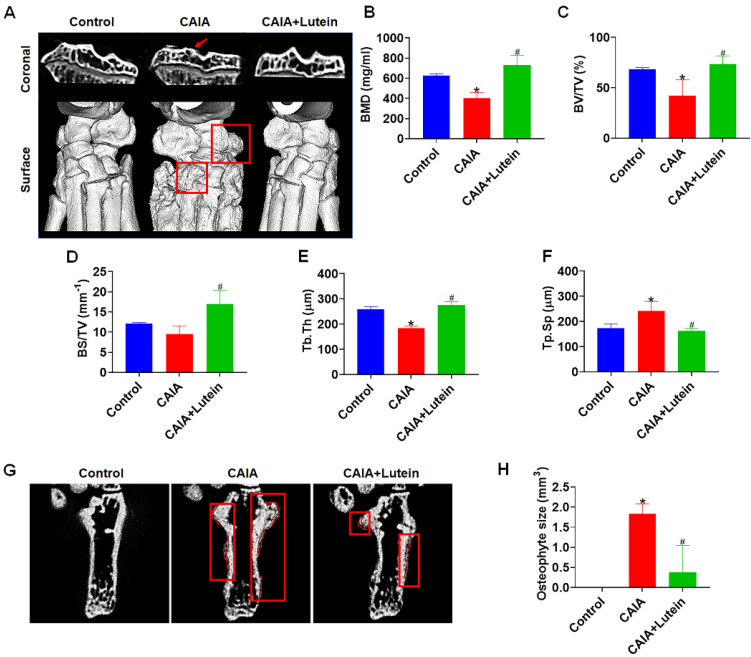
** Lutein reduces CAIA-induced bone erosion and osteophytes.** (A) Micro-CT representative images of the coronal part and surface of the bone. (B) Quantitative analyses of the bone mineral density (BMD). (C) Quantitative analyses of the bone volume/total volume (BV/TV). (D) Quantitative analyses of the bone surface/total volume (BS/TV). (E) Quantitative analyses of the trabecular thickness (Tb.Th). (F) Quantitative analyses of the trabecular separation (Tb.Sp). (G) Micro-CT representative images of the osteophyte formation. (H) Quantitative results of osteophyte volume. Untreated mice were used as controls. Results are shown as means ± SD. *p < 0.05 compared to untreated control. #*p* < 0.05 compared to CAIA.

Our micro-CT analysis provided a detailed examination of the effect of lutein on bone integrity in the context of CAIA. After sacrificing the mice, the hindlimbs were subjected to a comprehensive examination. The resulting data revealed that lutein significantly reduced the bone degeneration typically induced by CAIA. This was evidenced by the maintenance of bone mineral density (BMD) and the bone volume/tissue volume (BV/TV) ratio, which are critical indicators of bone health (Fig. 4A-C). Additionally, lutein administration increased bone surface/total volume (BS/TV) and trabecular thickness (Tb. Th), further supporting a protective effect against bone erosion (Fig. 4D-E). Notably, lutein also alleviated the CAIA-induced increase in trabecular separation (Tb. Sp), as observed in the CAIA lutein-treated group compared to the CAIA-alone group (Fig. 4F). Moreover, the formation of osteophytes, a hallmark of joint degeneration, was significantly reduced in the presence of lutein (Fig. 4G-H). These findings collectively demonstrate that lutein attenuates CAIA-induced bone destruction and morphological abnormalities, supporting a joint-protective effect in experimental inflammatory arthritis.

### Lutein Reduced Cartilage Destruction in CAIA Mice

**Figure 5:**
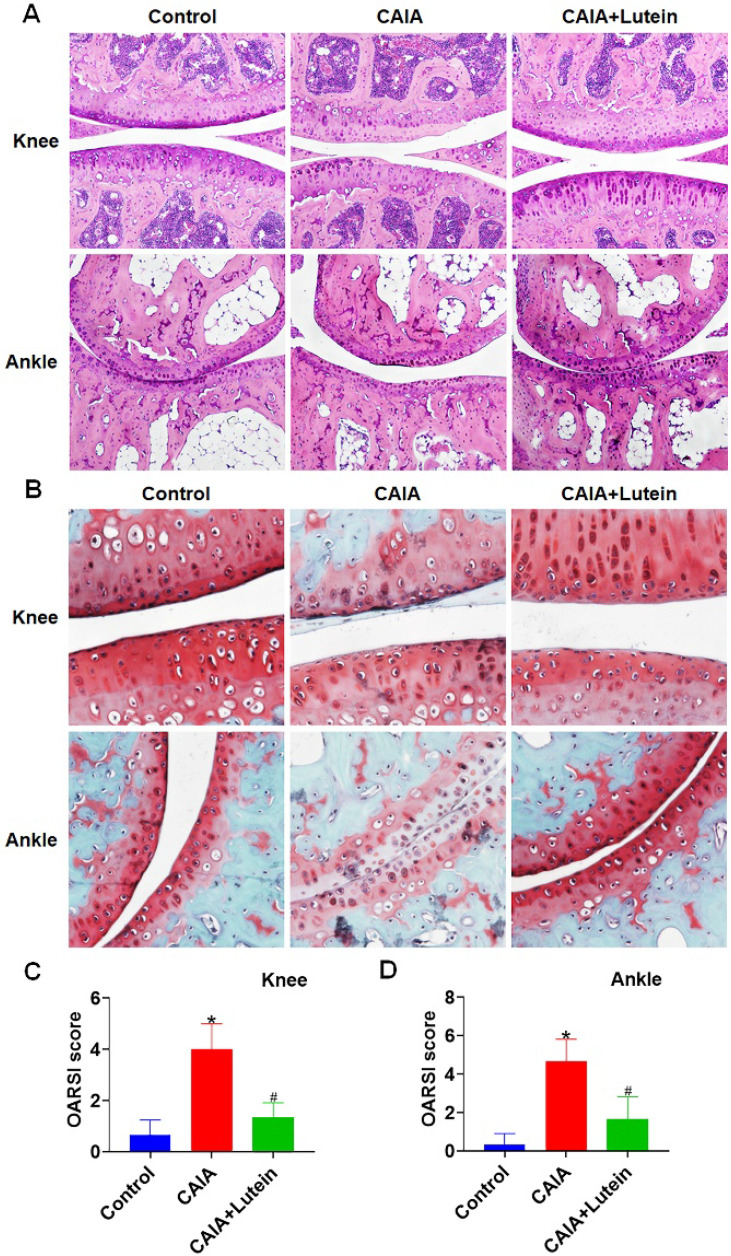
** Lutein reduces CAIA-induced cartilage degeneration.** (A) Representative images of hematoxylin and eosin (H&E) staining of mouse knee and ankle joint sections. (B) Representative Safranin O-stained sections from the knee and ankle joints of mice. (C)-(D) OARSI scores at the knee and ankle joints of mice. Untreated mice were used as controls. Results are shown as means ± SD. *p < 0.05 compared to untreated control. #*p* < 0.05 compared to CAIA.

To further investigate the protective effects of lutein on RA, histological examinations on mouse hindlimb tissue sections were conducted. Hematoxylin and eosin (H&E) staining showed that lutein administration mitigated the progression of cartilage surface irregularities induced by CAIA (Fig. 5A), which suggests a protective effect of lutein on cartilage. Subsequent examination with Safranin O staining confirmed these findings, revealing a deceleration in the degenerative changes of cartilage typically associated with CAIA following the administration of oral gavage lutein (Fig. 5B). The imaging data together with the Osteoarthritis Research Society International (OARSI) scoring system indicated a significant reduction in cartilage pathology (Fig. 5C-D). These findings support a protective effect of lutein against cartilage degeneration in the CAIA model.

### Lutein Reduced Synovium Thickening and Expression of Inflammatory Cytokines in CAIA Mice

**Figure 6:**
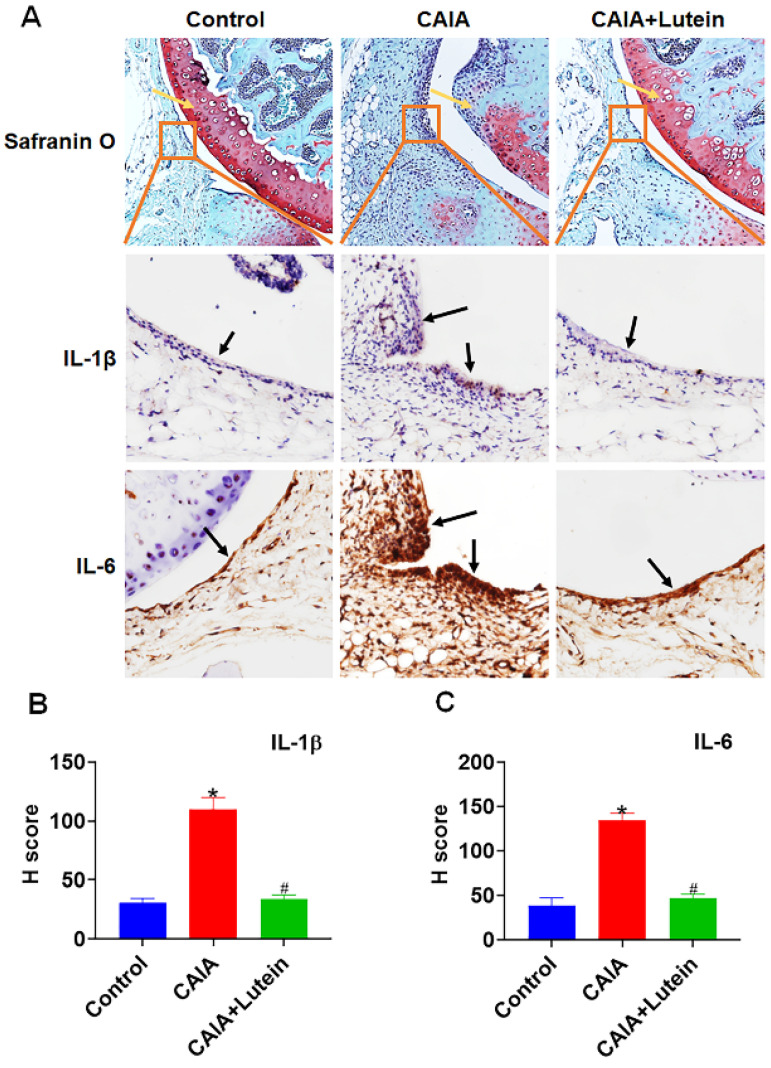
** Lutein reduces synovial inflammation induced by CAIA.** (A) Representative images of Safranin O staining and immunohistochemistry (IHC) staining of synovial sections around the knee of mice. (B)-(C) IHC staining analysis of IL-1β and IL-6 H scores. Untreated mice were used as controls. Results are shown as means ± SD. *p < 0.05 compared to untreated control. #*p* < 0.05 compared to CAIA.

Based on previous research linking synovial proliferation with an increase in pro-inflammatory cytokines 10-12, our IHC analysis further supported the anti-inflammatory potential of lutein in the context of RA. Safranin O-stained knee sections revealed that lutein administration effectively reduced the synovial membrane thickening and cartilage damage typically induced by CAIA (Fig. 6A). In support, quantification of IHC staining determined the impact of lutein on cytokine expression, in which the assessed H score indicated a significant decrease in the levels of IL-1β and IL-6 within the synovial tissue (Fig. 6A-C). These results support the hypothesis that lutein inhibits inflammatory cytokine expression and reduces synovial hyperplastic changes in experimental models relevant to RA.

## Discussion

RA is a debilitating autoimmune condition characterized by chronic joint inflammation and synovial hyperplasia, resulting in pain and bone erosion 1, 2. Although NSAIDs, DMARDs, and biologic therapies are available 3, 4, these medications cannot completely relieve symptoms and harbor numerous side effects 5, 6. These limitations highlight the need for alternative or complementary therapeutic approaches for RA. Lutein, a bioactive natural carotenoid with reported antioxidant and anti-inflammatory properties 21-26, has therefore attracted attention as a potential modulator of inflammatory disease. In the present study, RNA-seq analysis was used to characterize the transcriptomic effects of lutein in MH7A fibroblast-like synovial cells, and the *in vitro* findings were further supported by *in vivo* evidence showing attenuation of joint inflammation, cartilage damage, and bone erosion in the CAIA model.

The numerous protective effects of lutein include reducing oxidative damage to the lungs and liver 24 and inhibiting osteoporosis caused by osteoclast activation 25. Lutein is also significantly associated with the prevalence of cardiovascular disease and stroke 26. Patients with RA have lower plasma carotenoids and higher C-reactive protein levels 35; however, the exact mechanism and roles of these compounds in RA are not fully understood. The multifaceted progression of RA is characterized by the hyperactivation of fibroblast-like synovial cells. These cells proliferate and invade adjacent tissues, leading to the structural reorganization of joints and fostering an environment conducive to immune cell recruitment, all of which are hallmarks of RA pathology 14. Additionally, fibroblast-like synovial cell-induced neutrophil infiltration disrupts bone homeostasis, exacerbating joint degeneration and erosion 36. Various growth factors that are overexpressed in RA further worsen this condition by promoting pannus formation and causing irreversible joint damage 15, 16. In our current study, RNA-seq analysis was employed to elucidate the influence of lutein on these pathological processes. Our findings revealed that lutein downregulated genes enriched in inflammation-related biological processes, including acute inflammatory response, inflammatory response, regulation of interleukin-6 production, and regulation of cytokine production. The downregulated genes were also associated with cytokine-related molecular functions, such as cytokine activity, receptor ligand activity, and cytokine receptor binding. In addition, KEGG analysis highlighted rheumatoid arthritis, IL-17 signaling pathway, and cytokine-cytokine receptor interaction among the suppressed pathways. Moreover, GSEA further supported the suppressive effect of lutein on inflammation-related transcriptomic programs, including gene sets associated with interleukin-1 production, cytokine-mediated signaling, acute inflammatory response, and cytokine-cytokine receptor interaction. IL-1β and IL-6 were selected as representative validation markers because they are key inflammatory mediators in RA and are closely linked to the cytokine-related pathways highlighted by our RNA-seq, KEGG, and GSEA analyses. Together, these transcriptomic findings suggest that lutein modulates inflammatory pathways relevant to RA pathogenesis. Our *in vitro* experiments further showed that lutein inhibited synovial fibroblast proliferation and reduced the expression of inflammation-related cytokines in TNF-α-activated MH7A cells. These results support lutein as a bioactive natural compound with anti-inflammatory and joint-protective effects in experimental models relevant to RA. We also acknowledge that MH7A is an immortalized synoviocyte cell line and may not fully recapitulate the phenotype, heterogeneity, and disease-specific properties of primary RA fibroblast-like synoviocytes. Therefore, the present mechanistic findings should be interpreted with caution and would benefit from future validation in primary RA-FLS or other clinically relevant systems.

The CAIA model, which has been widely utilized in the literature 37, 38, served as our experimental model to assess the impact of lutein on RA-induced joint damage. In addition, because lutein administration was initiated before CAIA induction, the present *in vivo* design should be interpreted primarily as a preventive or early-intervention paradigm rather than a treatment model for established RA. Moreover, CAIA mainly reflects the antibody-mediated effector phase of arthritis and does not fully reproduce the chronic immune priming, adaptive autoimmunity, and clinical heterogeneity of human RA. We utilized OARSI indexes and histopathological scores from relevant literature to examine the impact of lutein in mitigating joint damage induced by RA. Micro-CT imaging and morphological observation confirmed the protective effects of lutein against CAIA-induced reductions in bone mineral density and trabecular integrity as well as the efficacy of lutein in diminishing abnormal osteophyte formation post-bone destruction. Notably, lutein significantly alleviated joint redness, swelling, and bone damage, further supporting its protective effects in experimental inflammatory arthritis. Although our CAIA control group displayed significant arthritis compared to the normal group, the degree of joint destruction observed was milder than in some previously reported CAIA models. This variability may stem from differences in experimental design, including the mouse strain used (Balb/c mice, which are reported to show less severe susceptibility compared to DBA/1 mice), lower antibody cocktail and LPS booster dose, and the disease kinetics, as joint pathology often peaks earlier (10-14 days) and may partially resolve by day 22 37-41. These factors likely account for the relatively modest histological changes and the lower OARSI scores we observed in the CAIA group. Importantly, despite this milder phenotype, our CAIA mice still demonstrated clear evidence of cartilage loss and reduced Safranin O staining compared to normal controls, and lutein treatment consistently attenuated these changes. Inter-study variability in CAIA severity has also been documented by others, highlighting that strain- and protocol-dependent differences can influence the extent of cartilage damage and scoring outcomes. These considerations should therefore be considered when interpreting our findings, and underscore that the protective effects of lutein remain evident even under a less aggressive disease phenotype. Studies have reported that the activation of synovial fibroblasts is highly related to the progression of RA 7-9. Through the proliferation of synovial fibroblasts, various inflammatory factors (TNF-α, IL-1β, and IL-6) are upregulated, thereby exacerbating the vicious cycle of inflammation, leading to irreversible joint destruction 10-13, 17-20. In our study, lutein alleviated CAIA-induced cartilage loss, as demonstrated by H&E and Safranin O staining, which was accompanied by reduced synovial hyperplasia and inflammatory changes in joint tissue. In addition to reducing synovial thickening, lutein also suppressed IL-1β and IL-6 expression in synovial tissue from CAIA mice, indicating attenuation of local inflammatory responses.

Although lutein showed anti-inflammatory and joint-protective effects in our experimental models, its translational relevance to human RA remains to be established. Given its established use as a dietary carotenoid, lutein may be considered for further mechanistic and preclinical evaluation as a candidate adjunctive bioactive compound in RA-related settings. Future studies should further define the optimal dosage, treatment duration, pharmacokinetic properties, oral bioavailability, achievable tissue exposure, and potential interactions of lutein with established anti-rheumatic therapies. Further validation in additional preclinical and translational models will be necessary to clarify the settings in which lutein may have the greatest biological relevance. These considerations support further investigation of lutein in well-designed mechanistic and preclinical studies, with additional translational evaluation required before clinical implications can be inferred.

## Conclusions

In summary, our findings demonstrate that lutein exerts anti-inflammatory and joint-protective effects *in vitro* and *in vivo*, attenuating inflammation-related responses in MH7A cells and reducing synovial inflammation, cartilage degradation, and bone destruction in the CAIA model. These results support further mechanistic and preclinical investigation of lutein in experimental models relevant to RA.

## Data Availability

The RNA-sequencing dataset generated in this study has been deposited in the NCBI Gene Expression Omnibus (GEO) under accession number GSE301891. The dataset will be made publicly available upon publication.

## Abbreviations

BMD: bone mineral density
BP: biological process
BS: bone surface
BV: bone volume
CAIA: collagen antibody-induced arthritis
CC: cellular component
DMARDs: disease-modifying antirheumatic drugs
FLS: fibroblast-like synovial cell
GEO: Gene Expression Omnibus
GO: gene ontology
GSEA: gene set enrichment analysis
IHC: Immunohistochemistry
IL-1β: interleukin-1β
IL-6: interleukin-6
KEGG: Kyoto Encyclopedia of Genes and Genomes
MF: molecular function
Micro-CT: computed tomography
MH7A: fibroblast-like synovial cell line
NSAIDs: nonsteroidal anti-inflammatory drugs
PBST: phosphate-buffered saline with Tween 20
RA: rheumatoid arthritis
RLE: relative log expression
RNA-seq: RNA-sequencing
RT-qPCR: real-time quantitative polymerase chain reaction
Tb. Th: trabecular thickness
TMM: trimmed mean of M-values
TNF-α: tumor necrosis factor-alpha
TV: tissue volume

## References

[1] Lee DM, Weinblatt ME (2001). Rheumatoid arthritis. Lancet.

[2] Schinnerling K, Rosas C, Soto L (2019). Humanized Mouse Models of Rheumatoid Arthritis for Studies on Immunopathogenesis and Preclinical Testing of Cell-Based Therapies. Front Immunol.

[3] Buch MH (2018). Defining refractory rheumatoid arthritis. Ann Rheum Dis.

[4] Nagy G, Roodenrijs NMT, Welsing PMJ (2022). EULAR points to consider for the management of difficult-to-treat rheumatoid arthritis. Ann Rheum Dis.

[5] Weyand CM, Goronzy JJ (2021). The immunology of rheumatoid arthritis. Nat Immunol.

[6] Braverman G, Bridges SL, Moreland LW (2022). Tapering biologic DMARDs in rheumatoid arthritis. Curr Opin Pharmacol.

[7] Aletaha D, Smolen JS (2018). Diagnosis and Management of Rheumatoid Arthritis: A Review. JAMA.

[8] da Fonseca LJS, Nunes-Souza V, Goulart MOF (2019). Oxidative Stress in Rheumatoid Arthritis: What the Future Might Hold regarding Novel Biomarkers and Add-On Therapies. Oxid Med Cell Longev.

[9] Qiu J, Wu B, Goodman SB (2021). Metabolic Control of Autoimmunity and Tissue Inflammation in Rheumatoid Arthritis. Front Immunol.

[10] Bartok B, Firestein GS (2010). Fibroblast-like synoviocytes: key effector cells in rheumatoid arthritis. Immunol Rev.

[11] Fox DA, Gizinski A, Morgan R (2010). Cell-cell interactions in rheumatoid arthritis synovium. Rheum Dis Clin North Am.

[12] Take Y, Nakata K, Hashimoto J (2009). Specifically modified osteopontin in rheumatoid arthritis fibroblast-like synoviocytes supports interaction with B cells and enhances production of interleukin-6. Arthritis Rheum.

[13] Kondo N, Kuroda T, Kobayashi D (2021). Cytokine Networks in the Pathogenesis of Rheumatoid Arthritis. Int J Mol Sci.

[14] Kugler M, Dellinger M, Kartnig F (2023). Cytokine-directed cellular cross-talk imprints synovial pathotypes in rheumatoid arthritis. Ann Rheum Dis.

[15] Raj R, Thomas S, Gorantla V (2022). Accelerated atherosclerosis in rheumatoid arthritis: a systematic review. F1000Res.

[16] Pamies A, Llop D, Ibarretxe D (2024). Angiopoietin-2, vascular endothelial growth factor family, and heparin binding endothelial growth factor are associated with subclinical atherosclerosis in rheumatoid arthritis. Comput Struct Biotechnol J.

[17] Bottini N, Firestein GS (2013). Duality of fibroblast-like synoviocytes in RA: passive responders and imprinted aggressors. Nat Rev Rheumatol.

[18] Sabeh F, Fox D, Weiss SJ (2010). Membrane-type I matrix metalloproteinase-dependent regulation of rheumatoid arthritis synoviocyte function. J Immunol.

[19] Chakrabarti S, Hore Z, Pattison LA (2020). Sensitization of knee-innervating sensory neurons by tumor necrosis factor-alpha-activated fibroblast-like synoviocytes: an in vitro, coculture model of inflammatory pain. Pain.

[20] Korb-Pap A, Bertrand J, Sherwood J (2016). Stable activation of fibroblasts in rheumatic arthritis-causes and consequences. Rheumatology (Oxford).

[21] Rutz JK, Borges CD, Zambiazi RC (2016). Elaboration of microparticles of carotenoids from natural and synthetic sources for applications in food. Food Chem.

[22] Ahn YJ, Kim H (2021). Lutein as a Modulator of Oxidative Stress-Mediated Inflammatory Diseases. Antioxidants (Basel).

[23] Pap R, Pandur E, Janosa G (2022). Lutein Decreases Inflammation and Oxidative Stress and Prevents Iron Accumulation and Lipid Peroxidation at Glutamate-Induced Neurotoxicity. Antioxidants (Basel).

[24] El-Kholy AA, Elkablawy MA, El-Agamy DS (2017). Lutein mitigates cyclophosphamide induced lung and liver injury via NF-kappaB/MAPK dependent mechanism. Biomed Pharmacother.

[25] Li H, Huang C, Zhu J (2018). Lutein Suppresses Oxidative Stress and Inflammation by Nrf2 Activation in an Osteoporosis Rat Model. Med Sci Monit.

[26] Wang M, Tang R, Zhou R (2023). The protective effect of serum carotenoids on cardiovascular disease: a cross-sectional study from the general US adult population. Front Nutr.

[27] Chang CJ, Lin JF, Chang HH (2013). Lutein protects against methotrexate-induced and reactive oxygen species-mediated apoptotic cell injury of IEC-6 cells. PLoS One.

[28] Qiao YQ, Jiang PF, Gao YZ (2018). Lutein prevents osteoarthritis through Nrf2 activation and downregulation of inflammation. Arch Med Sci.

[29] Szklarczyk D, Kirsch R, Koutrouli M (2023). The STRING database in 2023: protein-protein association networks and functional enrichment analyses for any sequenced genome of interest. Nucleic Acids Res.

[30] Park JS, Lee D, Yang S (2022). Methotrexate-loaded nanoparticles ameliorate experimental model of autoimmune arthritis by regulating the balance of interleukin-17-producing T cells and regulatory T cells. J Transl Med.

[31] Pietrosimone KM, Jin M, Poston B (2015). Collagen-Induced Arthritis: A model for Murine Autoimmune Arthritis. Bio Protoc.

[32] Luan J, Hu Z, Cheng J (2021). Applicability and implementation of the collagen-induced arthritis mouse model, including protocols (Review). Exp Ther Med.

[33] Domander R, Felder AA, Doube M (2021). BoneJ2 - refactoring established research software. Wellcome Open Res.

[34] Liu H, Ding J, Wang J (2015). Remission of collagen-induced arthritis through combination therapy of microfracture and transplantation of thermogel-encapsulated bone marrow mesenchymal stem cells. PLoS One.

[35] De Pablo P, Dietrich T, Karlson EW (2007). Antioxidants and other novel cardiovascular risk factors in subjects with rheumatoid arthritis in a large population sample. Arthritis Rheum.

[36] Carmona-Rivera C, Kaplan MJ, O'Neil LJ (2024). Neutrophils in Inflammatory Bone Diseases. Curr Osteoporos Rep.

[37] Irrera N, D'Ascola A, Pallio G (2019). beta-Caryophyllene Mitigates Collagen Antibody Induced Arthritis (CAIA) in Mice Through a Cross-Talk between CB2 and PPAR-gamma Receptors. Biomolecules.

[38] Jin CH, So Y, Nam B (2017). Isoegomaketone Alleviates the Development of Collagen Antibody-Induced Arthritis in Male Balb/c Mice. Molecules.

[39] Balkrishna A, Sakat SS, Joshi K (2019). Anti-Inflammatory and Anti-Arthritic Efficacies of an Indian Traditional Herbo-Mineral Medicine "Divya Amvatari Ras" in Collagen Antibody-Induced Arthritis (CAIA) Mouse Model Through Modulation of IL-6/IL-1beta/TNF-alpha/NFkappaB Signaling. Front Pharmacol.

[40] Stump KL, Lu LD, Dobrzanski P (2011). A highly selective, orally active inhibitor of Janus kinase 2, CEP-33779, ablates disease in two mouse models of rheumatoid arthritis. Arthritis Res Ther.

[41] van der Velden D, Lagraauw HM, Wezel A (2016). Mast cell depletion in the preclinical phase of collagen-induced arthritis reduces clinical outcome by lowering the inflammatory cytokine profile. Arthritis Res Ther.

